# Highly Structure‐Selective On‐Surface Synthesis of Isokekulene Versus Kekulene

**DOI:** 10.1002/anie.202509932

**Published:** 2025-07-29

**Authors:** Zilin Ruan, Qitang Fan, Alexander Reichmann, Faming Kang, Tim Naumann, Simon Werner, Olaf Kleykamp, Jose Martinez‐Castro, Felix Lüpke, Anja Haags, François C. Bocquet, Christian Kumpf, Serguei Soubatch, Jörg Sundermeyer, Peter Puschnig, F. Stefan Tautz, J. Michael Gottfried, Sabine Wenzel

**Affiliations:** ^1^ Department of Chemistry Marburg University 35037 Marburg Germany; ^2^ Hefei National Research Center for Physical Sciences at the Micro scale, Synergetic Innovation Center of Quantum Information & Quantum Physics, and New Cornerstone Science Laboratory University of Science and Technology of China Hefei Anhui 230026 China; ^3^ Institute of Physics, University of Graz, NAWI Graz Graz 8010 Austria; ^4^ Peter Grünberg Institute (PGI‐3), Forschungszentrum Jülich 52425 Jülich Germany; ^5^ Jülich Aachen Research Alliance (JARA) Fundamentals of Future Information Technology 52425 Jülich Germany; ^6^ Experimental Physics II B RWTH Aachen University 52074 Aachen Germany; ^7^ II. Physikalisches Institut, Universität zu Köln 50937 Köln Germany; ^8^ Experimental Physics IV A RWTH Aachen University 52074 Aachen Germany; ^9^ Present address: Chair of Physical Metallurgy University of Leoben Leoben 8700 Austria

**Keywords:** Aromaticity, Dehydrogenation, Scanning probe microscopy, Selectivity, Surface chemistry

## Abstract

The role of different facets of metal nanoparticles in steering reaction pathways is crucial for the design of heterogeneous catalysts with superior selectivity. As a prominent class of reactions, transition‐metal‐catalyzed carbon‐hydrogen (C─H) bond activation is widely used for the synthesis of base chemicals, modern organic materials, and pharmaceuticals. Here, we report orthogonal selectivity in intramolecular cyclodehydrogenation of a nonplanar cyclic precursor steered by different facets of a copper single crystal. On the Cu(110) surface, the previously unknown cycloarene isokekulene forms with a high selectivity of 92%, whereas reaction on the Cu(111) surface exclusively yields kekulene (>99%). Combining scanning tunneling microscopy with CO‐functionalized tips and density functional theory, we identify two adsorption geometries of the precursor, which react to the respective products. Isokekulene adopts two nonplanar adsorption configurations and exhibits strong molecule‐substrate interactions, explaining its preferential formation on Cu(110). This combined in‐solution and on‐surface synthesis approach represents an alternative route for the highly selective synthesis of molecules that are challenging to synthesize and process via conventional methods.

## Graphical abstract

On‐surface synthesis has allowed for the preparation of many novel molecular structures with intriguing properties. Here, we present a case of highly selective on‐surface synthesis of the previously unknown cycloarene isokekulene on Cu(110) and the prototypical cycloarene kekulene on Cu(111) from the very same specialized precursor prepared in solution.

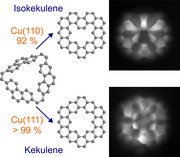

## Introduction

The production of base chemicals^[^ ^1^ ^]^ and industrial pharmaceuticals,^[^ ^2^ ^]^ as well as modern organic materials such as liquid crystals^[^ ^3^ ^]^ and conducting polymers,^[^ ^4^ ^]^ relies heavily on organic synthesis reactions catalyzed by transition metals. There has been increasing interest in using heterogeneous catalysts, in particular in the form of metal nanoparticles, because they are often more sustainable.^[^ ^1^, ^5^ ^]^ Their catalytic properties are not only highly dependent on the choice of the metal but also on the size, shape, and crystal facets of the particles.^[^ ^6^, ^7^ ^]^ Different metals and facets can be compared in controlled surface‐science studies on distinct, well‐defined faces of single crystals.^[^ ^8^ ^]^ In recent years, the on‐surface synthesis of polycyclic aromatic hydrocarbons (PAHs) by cyclodehydrogenation on single crystals of Cu, Ag, Au, and Pt has been studied extensively, allowing the successful synthesis of graphene nanoribbons,^[^ ^9^, ^10^, ^11^, ^12^, ^13^, ^14^, ^15^ ^]^ nanographenes,^[^ ^16^, ^17^, ^18^, ^19^, ^20^, ^21^, ^22^, ^23^, ^24^ ^]^ and, very recently, graphene nanorings or cycloarenes.^[^ ^25^, ^26^, ^27^, ^28^, ^29^ ^]^ The nature of the metal and the crystallographic surface orientation can influence the product composition. The most prominent example of such selectivity are nanoribbons with different edge structures and smaller nanographenes formed from the same precursor molecules.^[^ ^15^, ^30^, ^31^, ^32^, ^33^ ^]^ In these previous studies, the selectivity is due to different *inter*molecular Ullmann coupling and cyclodehydrogenation reactions between multiple precursor molecules depending on the chosen surface.

**Scheme 1:**
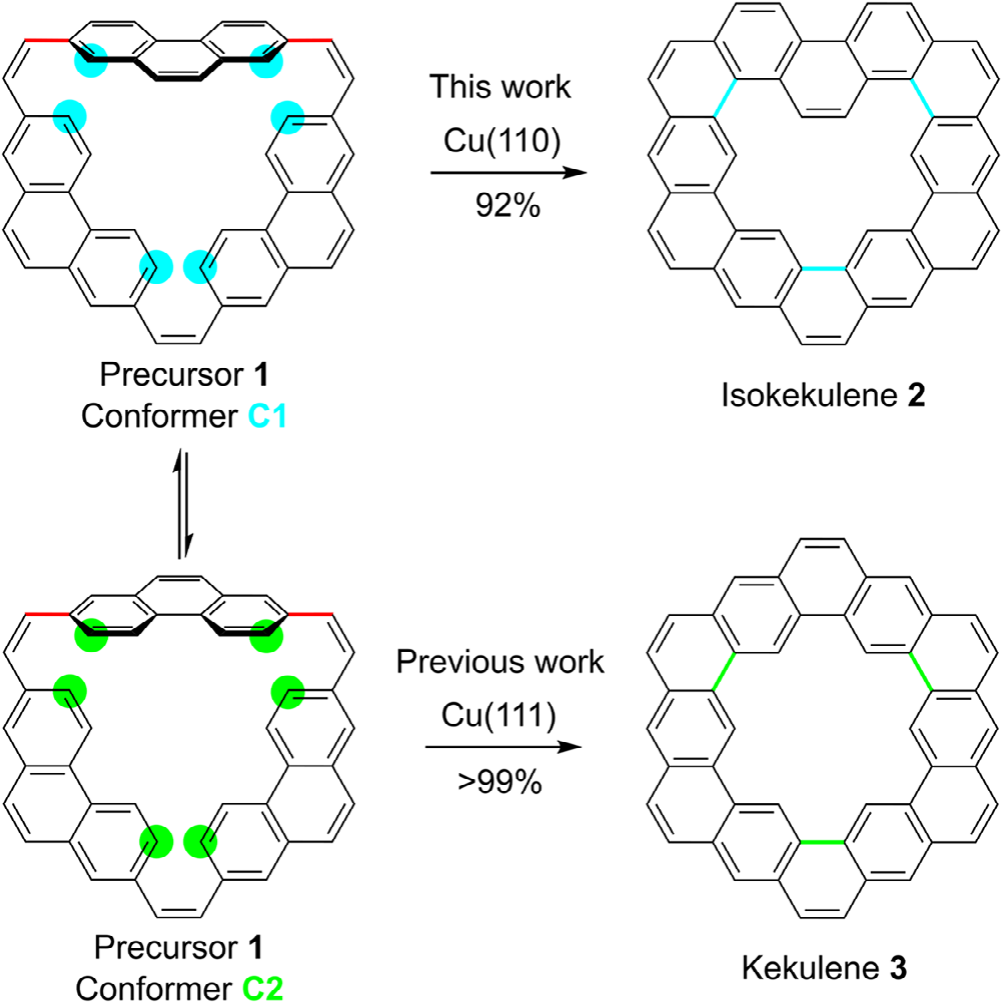
Reaction scheme for the precursor **1** on the Cu(110) and Cu(111) surfaces. In the adsorbed state, **1** forms the two different conformers **C1** and **C2**, which differ by a rotation of the top phenanthrene unit around the two σ bonds marked in red. These conformers are highly flexible and mobile at a reaction temperature of 500 K. The cyclodehydrogenation of **1** leads to the formation of isokekulene **2** (selectivity 92%) on Cu(110) and kekulene **3** (selectivity > 99%) on Cu(111).^[^ ^28^ ^]^ Cyan and green circles mark the carbon atoms between which the new carbon‐carbon bonds in the respective color are formed during the reaction.

Here, we report high selectivity at monolayer coverage in the formation of a previously unknown cycloarene, which we refer to as isokekulene **2** (see Scheme 1). The reaction proceeds via cyclodehydrogenation of the nonplanar cyclic precursor 1,4,7(2,7)‐triphenanthrena‐cyclononaphane‐2,5,8‐triene (Scheme 1, precursor **1**). On the reactive Cu(110) surface, we achieve a selectivity of up to 92% of isokekulene and a large yield of up to 85% surface coverage from a monolayer of the precursor. In contrast, the same precursor reacts on Cu(111) with a previously reported selectivity of 99% toward a full monolayer of kekulene **3**.^[^ ^28^ ^]^ To our knowledge, this represents the first case of different *intra*molecular cyclodehydrogenation reactions occurring within the same precursor molecule, catalyzed by structurally distinct facets of the same metal.

## Results and Discussion

**Figure 1:**
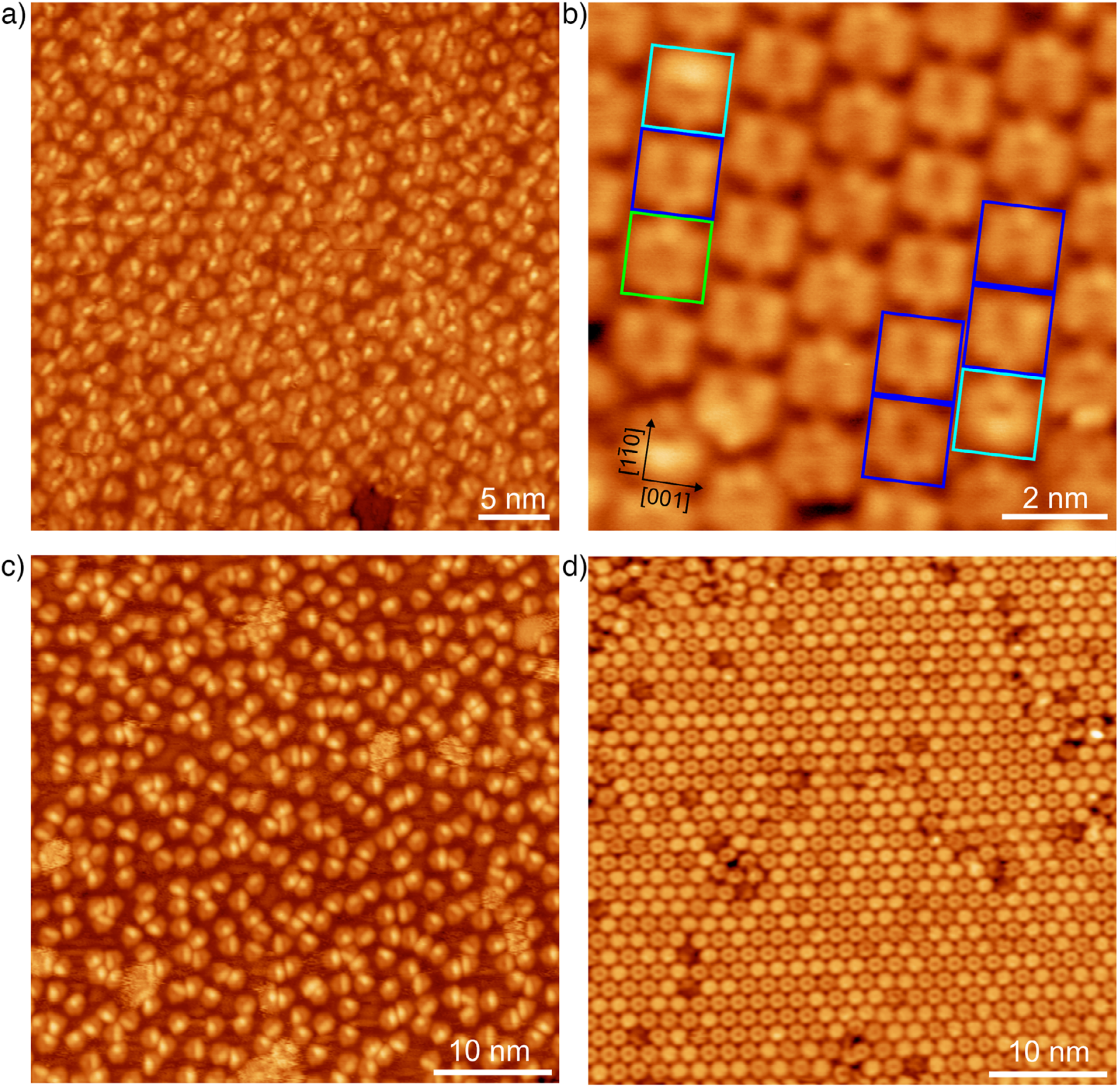
a) Overview STM image of a monolayer of the precursor **1** on Cu(110) and b) smaller‐scale STM image of the product molecules formed by annealing the same surface to 500 K. Green, cyan, and dark blue squares in (b) mark kekulene molecules, isokekulene molecules in the up configuration, and isokekulene molecules in the down configuration, respectively. c) Overview STM image of a monolayer of the precursor **1** on Cu(111) and d) STM image of the kekulene product after annealing to 500 K. The tunneling parameters are (a) *U =* 1.7 V, *I =* 80 pA, (b) *U* *=* −1.0 V, *I =* 210 pA, (c) *U =* 3.0 V, *I =* 80 pA, and (d) *U* *=* −3.0 V, *I =* 350 pA. All images are taken at 100 K surface temperature.

**Figure 2:**
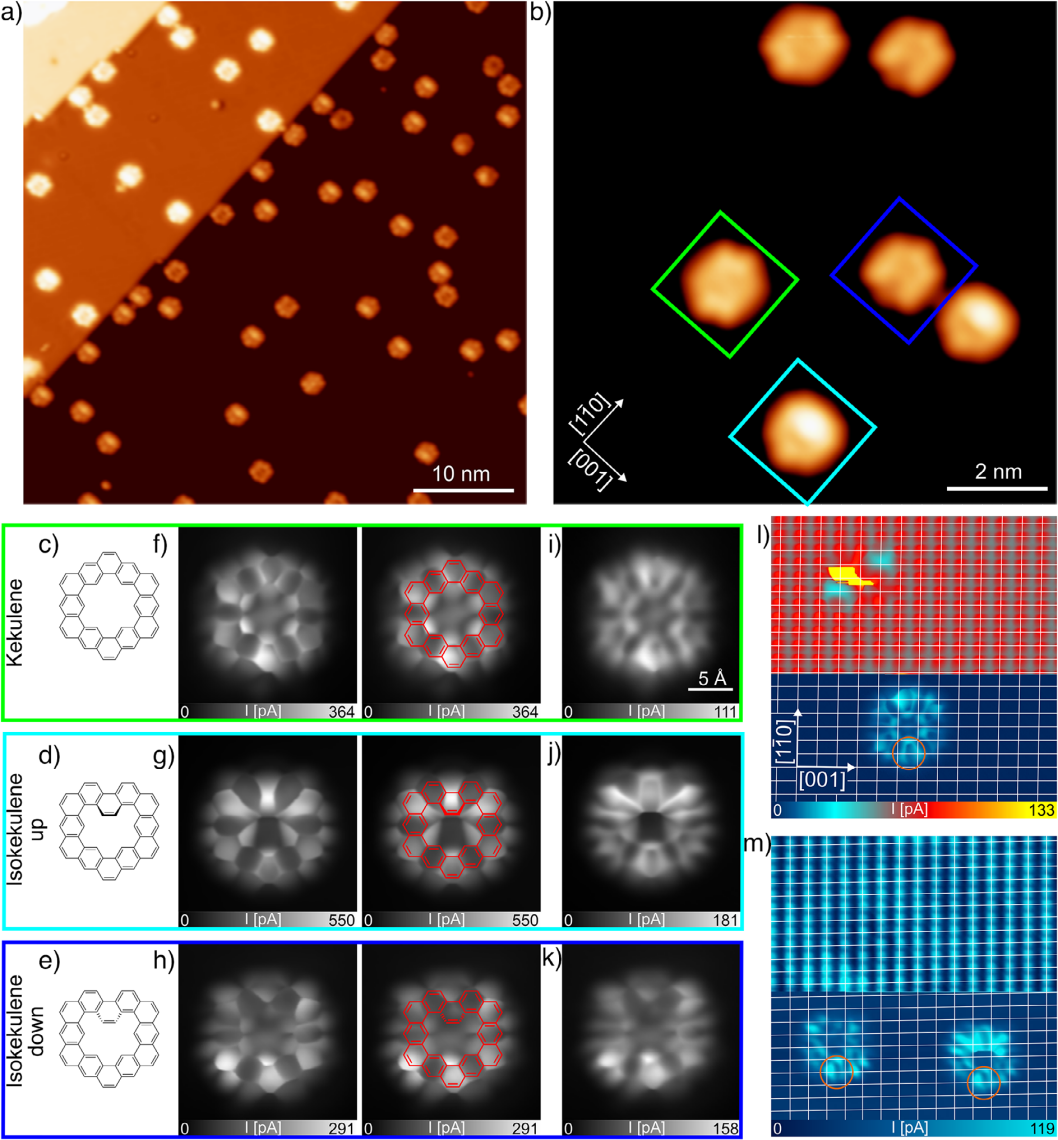
a) Overview constant‐current STM image (*U =* 90 mV, *I =* 20 pA) taken after annealing a low coverage of precursor **1** on the Cu(110) surface to 500 K. b) Smaller‐scale constant‐current STM image (*U =* 100 mV, *I = *80 pA) of the formed products. The frames mark examples of isokekulene in the up configuration (cyan) and the down configuration (dark blue) as well as kekulene (green). The corresponding chemical structures are shown in c)–e). f)–k) Constant‐height STM images of the three species recorded with a CO‐functionalized tip (f–h) at a low distance with and without the chemical structure overlaid, as well as (i–k) at a larger distance for comparison. Hereby, the height of the tip was initially set by the feedback above the substrate at *U =* 5 mV and *I = *20 pA. Subsequently, with the feedback turned off, the height was increased by (f) *z =* 50 pm, (g) 80 pm, (h) 60 pm, (i) 100 pm, (j) 125 pm, and (k) 90 pm. l) and m) Constant‐height STM images, in which (l) kekulene as well as (m) isokekulene in the (left) down and (right) up configuration are visible in the bottom part, whereas the copper surface atoms are resolved in the top part. The atomic resolution is scanned at the initial height, whereas the molecules are imaged roughly 200 pm above this position. White lines mark the unit cell and orange circles show the bottom part of the molecules, which appears the same for all three species. All images are taken at 4 K surface temperature.

Figure 1a shows a scanning tunneling microscopy (STM) image taken after depositing precursor **1** onto the Cu(110) surface at 300 K, which leads to a disordered adsorbed layer of approximately monolayer coverage. Upon annealing to 500 K, the precursor reacts to form well‐ordered, close‐packed domains of cyclodehydrogenated products, as shown in Figure 1b. Unlike the precursor, the products show a preferred orientation aligned with the $\left[1\bar{1}0\right]$‐direction of the Cu(110) substrate. Close inspection reveals three different species marked by the green, cyan, and dark blue frames in Figure 1b. The green‐framed species has a regular hexagonal shape, consistent with the molecular backbone of kekulene (**3** in Scheme 1), which has been synthesized previously in solution^[^ ^34^, ^35^ ^]^ and via on‐surface synthesis methods.^[^ ^28^ ^]^ In contrast, the cyan and dark blue‐framed species have an approximately pentagonal shape, resembling a hexagon with one missing corner, instead of which a bright protrusion or a (faint) depression is observed. This product is tentatively attributed to isokekulene (**2** in Scheme 1), which is described here for the first time. Compared to kekulene, one of the three phenanthrene moieties in isokekulene is flipped such that its central benzene ring points inwards, i.e., into the central pore. As this pore is too small to accommodate all hydrogen atoms facing toward the center of the molecule, isokekulene is nonplanar with the central benzene ring pointing either away from or toward the surface. The bright feature of the molecule in the cyan frame in Figure 1b indicates that this molecule has an “up” configuration. The more even contrast of the dark blue‐framed molecule indicates that this molecule has a “down” configuration (see the chemical structures in Figure 2d,e).

Overall, isokekulene is formed with a high selectivity, as indicated by its large fraction of 92% in the product mixture. (The different molecules are marked in two example images in Figure S1 in the Supporting Information.) Excluding some molecules which cannot clearly be identified, this corresponds to a surface coverage with isokekulene molecules of at least 85%.

Deposition of the same precursor onto the Cu(111) surface leads to a comparable disordered monolayer (compare Figure 1c to 1a). However, after annealing to 500 K, exclusively kekulene molecules are found in an ordered monolayer (Figure 1d), in agreement with previous work.^[^ ^28^ ^]^

To validate the identification of the three different molecular species described in the previous section, high‐resolution STM imaging was performed at 4 K. For this, a low coverage of precursor **1** was deposited onto the Cu(110) surface at 300 K and subsequently annealed to 500 K. Figure 2a shows a large‐scale overview image of the resulting isolated product molecules, which are still aligned with the high‐symmetry direction of the substrate. The smaller‐scale image in Figure 2b, obtained with a metal tip, reveals the three different molecular contrasts, consistent with the full coverage case presented in Figure 1b.

The STM images of the three product species presented in Figure 2f–k were recorded with a CO‐functionalized tip. This allows for a higher resolution due to two distinct processes: First, tunneling contributions from and to *p*‐wave orbitals of the CO lead to an STM contrast related to the lateral gradient of the wave function instead of the wave function as for conventional STM tips.^[^ ^36^ ^]^ Second, specific interactions with the adsorbed molecules lead to reversible movements of functionalized tips during the scanning motion, which typically causes specific, sharp contrasts. In the following, we refer to the different STM contrast due to these phenomena as geometric‐type and electronic‐type contrast, respectively. As opposed to the electronic‐type contrast, the geometric‐type contrast is also present at bias voltages within the HOMO‐LUMO gap. It allows for clear structural identification of adsorbed molecules.^[^ ^37^, ^38^, ^39^ ^]^ At low tip heights, this type of imaging is dominant, as visible in the STM images presented in Figure 2f–h. Therefore, the twelve benzene rings of kekulene can be clearly recognized (Figure 2f). The rings appear alternatingly bright and dark, with the Clar sextets of kekulene^[^ ^28^ ^]^ being dark. Due to the 2‐fold symmetry of the substrate, the molecule appears somewhat elongated in the vertical direction of the image, which is parallel to the $\left[1\bar{1}0\right]$‐direction, as opposed to the 6‐fold symmetry it has on Cu(111). This is in agreement with the symmetry of the kekulene molecule as imaged with a metallic tip on the monolayer as well as the low coverage surface (see Figures 1b and 2b, respectively). The high‐resolution image of isokekulene in the up configuration (Figure 2g) clearly shows the central benzene ring in its pore pointing upwards as well as the resulting pentagonal shape. Due to the two up‐facing hydrogen atoms, the molecule could not be approached as closely as the kekulene molecule without strong interaction between the tip and the molecule leading to distortions in the image. Some geometric‐type imaging is still visible at a distance of *z =* 125 pm (see Figure 2j) only at the highest part of the molecule, whereas the bottom part shows the electronic‐type STM contrast. On the other hand, isokekulene molecules in the down configuration (see Figure 2h) can be approached more closely with the tip, but the down‐facing benzene ring cannot be made as clearly visible without the tip interacting too strongly with the rest of the molecule. Still, the two benzene rings left and right of the central one can be distinguished, and the overall pentagonal shape is clearly visible. Comparing the contrast of the single benzene rings of isokekulene to kekulene suggests that the Clar sextets are mainly located in the same benzene rings of all three molecules as is indicated by the structures overlaid on the right side of Figure 2f–h. The geometric‐type contrast of all three molecules is surrounded by less intense features, which suggests an additional electronic‐type contrast.^[^ ^40^ ^]^ The electronic‐type contrast at the peripheries is in agreement with the STM contrast presented in Figure 2i–k. These images are taken at rather low voltages of 5 mV, but at a larger tip‐sample distance where the electronic‐type imaging is dominant. As no electronic states are expected close to the Fermi energy for the isolated molecules in the gas phase, it is likely that these are due to hybridization between molecular and surface electronic states. This is typically indicative of a strong molecule‐metal interaction,^[^ ^41^ ^]^ which will be discussed below.

In order to identify the adsorption sites of the product molecules, the STM images in Figure 2l,m were recorded, in which molecules of each species are resolved in the bottom part, whereas the Cu(110) surface is atomically resolved in the top part of the images.^[^ ^42^ ^]^ This is achieved by changing the tip height while scanning (see the figure caption for details). Equidistant white lines were then drawn on top of the image through the copper atoms in the high‐symmetry directions of the substrate, i.e., horizontally parallel to the [001]‐direction and vertically parallel to the $\left[1\bar{1}0\right]$‐direction. For the kekulene molecule (see Figure 2l), one can clearly see that one of the horizontal lines passes directly through the center of its pore, which agrees well with the adsorption geometry at the long bridge site (see the theoretical geometry presented in Figure 4a). In contrast to kekulene, the isokekulene molecule (see one molecule in the down configuration on the left side and one in the up configuration on the right side of Figure 2m) is not mirror symmetric with respect to the [001]‐direction. Therefore, it is more challenging to distinguish whether one of the horizontal lines passes through the center of the pore. However, the bottom part of the molecules, which is identical for all three species, appears to be positioned in the same way with respect to the copper atoms. This is indicated by the orange circles in Figure 2l,m. As no significant statistics using a number of different tips can be achieved due to the time‐intensity of these measurements, thorough care has been taken to ensure the stability of the CO‐tip and drift corrections are implemented prior to recording these images. Thus, we consider it likely that, at a low coverage, all three species prefer the long bridge site. As shown below, this long bridge site is predicted as the favorable site by density functional theory (DFT) for kekulene and isokekulene in the up configuration.

**Figure 3:**
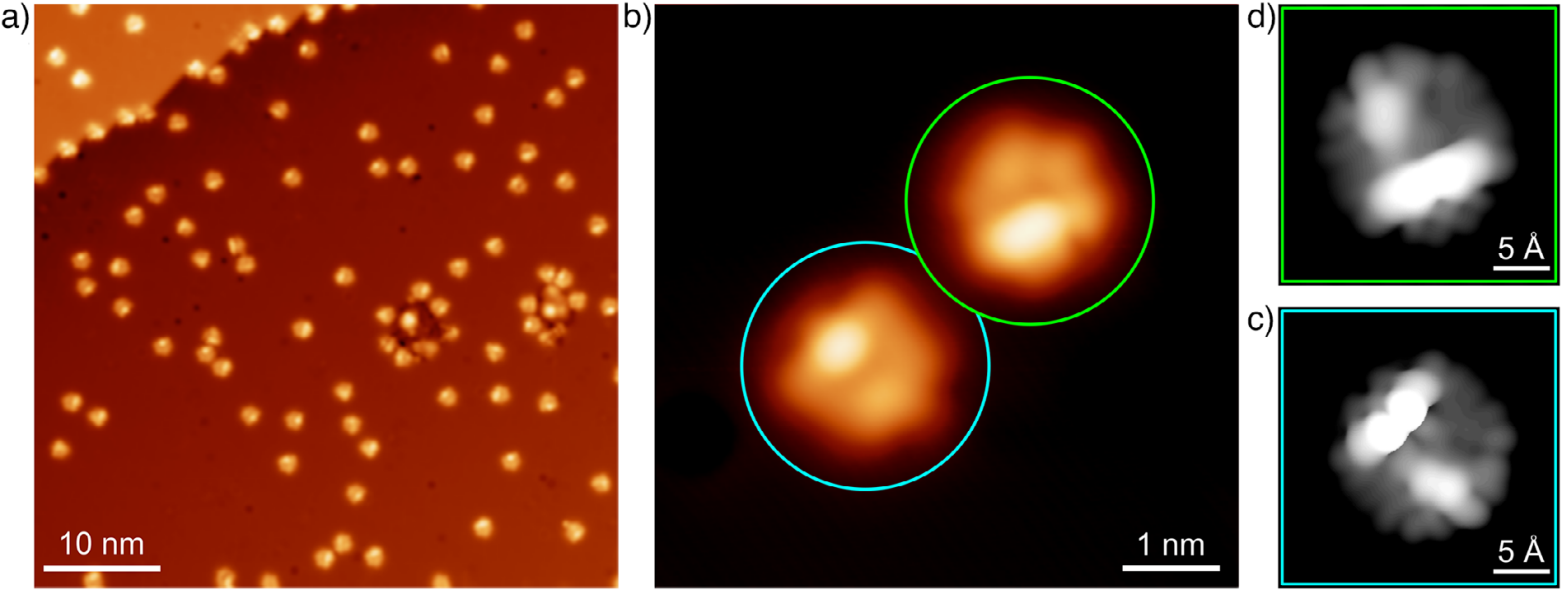
a) Overview STM image taken after deposition of a low coverage of precursor **1** onto the Cu(110) surface at 300 K. b) Smaller‐scale STM image of the same surface showing two intact precursor molecules. One is identified as conformer **C1** (cyan circle) and the other as conformer **C2** (green circle). The tunneling parameters are (a) *U =* 120 mV, *I =* 40 pA, and (b) *U* *=* –120 mV, *I =* 100 pA. The measurements are compared to simulated STM images of c) conformer **C1** and d) conformer **C2** based on the DFT geometries presented below.

The low‐coverage sample also allows for a more detailed structural investigation of the precursor **1** itself. Figure 3a shows an STM image taken after depositing a low coverage of **1** onto the Cu(110) surface at 300 K, resulting in isolated molecules distributed randomly over the surface. As seen in the smaller‐scale STM image in Figure 3b, two distinct molecular contrasts related to different species are observed, marked by the cyan and green circles. The cyan‐circled species has an irregular pentagonal shape with only one nearly circular bright protrusion in the center of the longest edge of the pentagon. We identify this species as a conformer of precursor **1**, in which one phenanthrene moiety is tilted so that its central benzene ring faces inward toward the center of the molecule and away from the surface, as illustrated by the chemical structure drawn in Scheme 1 (conformer **C1**) as well as the theoretical geometry shown in Figure 5a. In contrast, the green‐circled species displays a more elongated bright protrusion along the longest edge of the pentagon. This protrusion is attributed to the two outer benzene rings of the phenanthrene moiety when the central benzene ring points to the outside of the molecule and toward the surface. This conformer is denoted as **C2** (see also Scheme 1 and Figure 5b).

Due to the strongly nonplanar adsorption geometries, bond‐resolved imaging of the precursor is not feasible. Instead, the identification of the two conformers is confirmed by comparison with the simulated STM images in Figure 3c,d, which are based on the DFT calculations presented below. Notably, the structures of conformers **C1** and **C2,** as drawn in Scheme 1, exhibit mirror symmetry with respect to a vertical line. However, this symmetry appears not strictly fulfilled in the STM images, a feature more clearly visible in the simulated images. The relaxed DFT geometries of both conformers, in their most favorable adsorption site on Cu(110), reveal that one of the two bottom phenanthrene units (the left one in Figure 5a,b) is slightly tilted away from the surface, while only one of the three units (the right bottom one) can lie completely flat. Overall, this distortion highlights the steric hindrance between the two hydrogen atoms facing each other at each of the three junctions where two phenanthrene units meet—precisely where the reaction eventually takes place (see Scheme 1).

**Figure 4:**
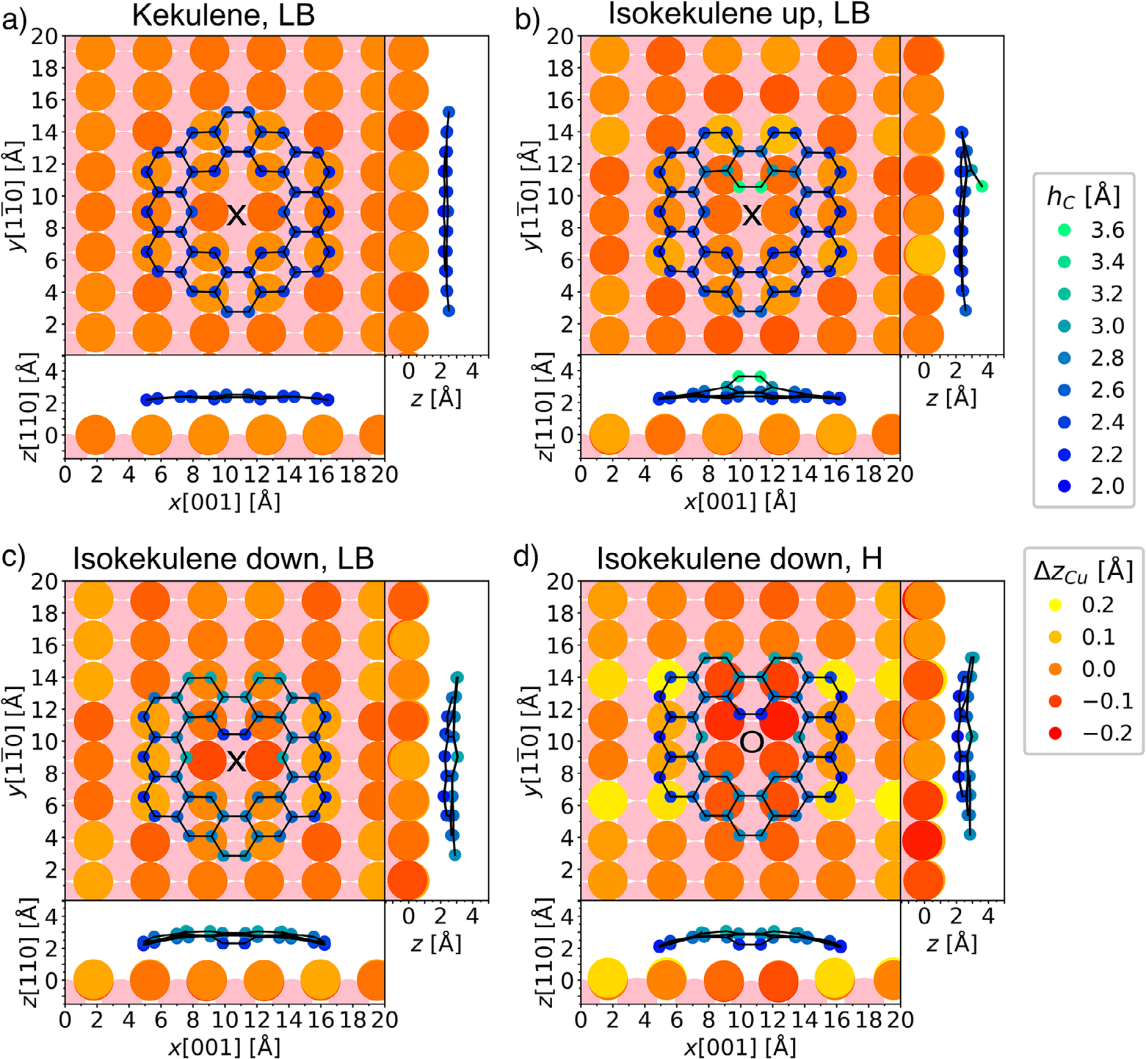
Calculated adsorption geometries of a) kekulene, b) isokekulene in the up configuration, and c) isokekulene in the down configuration at the long bridge (LB) site as well as d) the down configuration at the hollow (H) site of Cu(110). The calculations were performed using a five‐layer slab. The color scales show the adsorption height of the carbon atoms *h_C_* (with respect to the center of the topmost copper layer) in blue to green and the relaxation of the top layer of copper atoms *Δz_Cu_* in red to yellow. A second layer of copper atoms is displayed in light pink. For clarity, only one molecule is shown for each geometry, whereas those from neighboring unit cells are omitted. The PBE‐GGA functional combined with Tkatchenko–Scheffler dispersion corrections^[^ ^47^ ^]^ was used.

For a more detailed understanding of the geometric structure and the interaction of the molecular species with the copper surfaces, all relevant molecules were investigated using density functional theory. We start with the product molecules on Cu(110), comparing them to kekulene on Cu(111), which has been investigated previously.^[^ ^28^ ^]^ For this, the VASP code^[^ ^43^, ^44^, ^45^, ^46^ ^]^ was employed on repeated‐slab models of Cu. The core electrons were treated with the projector augmented wave method,^[^ ^43^, ^44^, ^45^, ^46^ ^]^ and the PBE‐GGA functional combined with dispersion corrections^[^ ^47^, ^48^ ^]^ was used (see Methods Section for details). With this approach, we have determined the most favorable adsorption geometries of kekulene and isokekulene in the up and down configurations on Cu(110). For isokekulene, a unit cell based on low‐energy electron diffraction (LEED) data of the full monolayer on Cu(110) can be used. As shown in Figure S4, the LEED measurement results in unit cell vectors of *a_1_ =* 1.6 nm and *a_2_ =* 1.5 nm at an angle of *γ* *=* 118°. In the case of kekulene, a larger unit cell is required. The resulting geometries are presented in Figure 4a–d, with corresponding energies summarized in Table S1. For kekulene as well as for isokekulene in the up configuration, the long bridge site, indicated with the black cross, is favorable with an energy benefit of 0.51 eV and 0.42 eV, respectively, over the second‐most stable hollow site. In contrast, isokekulene in the down configuration is predicted to be most stable in the hollow site (black circle in Figure 4d), where its energy is 0.52 eV lower than in the long bridge site. In both the long bridge and the hollow sites, four rows of Cu atoms contribute to the bonding of the molecules (as opposed to only three rows for the short bridge and top sites, not shown) thereby enabling a stronger interaction.

For all three species, DFT calculations yield adsorption heights that are smaller than the sum of the van der Waals radii of carbon and copper, indicating a chemical interaction with the Cu(110) surface. The adsorption height of kekulene, determined here to be 2.41 Å, can be directly compared to the height of 3.05 Å found on Cu(111),^[^ ^28^ ^]^ which confirms a stronger interaction with the more open Cu(110) surface. This is consistent with the STM contrast observed close to the Fermi energy in Figure 2i–k, which suggests the absence of a clear HOMO‐LUMO gap and hybridization between molecular and surface states.

**Figure 5:**
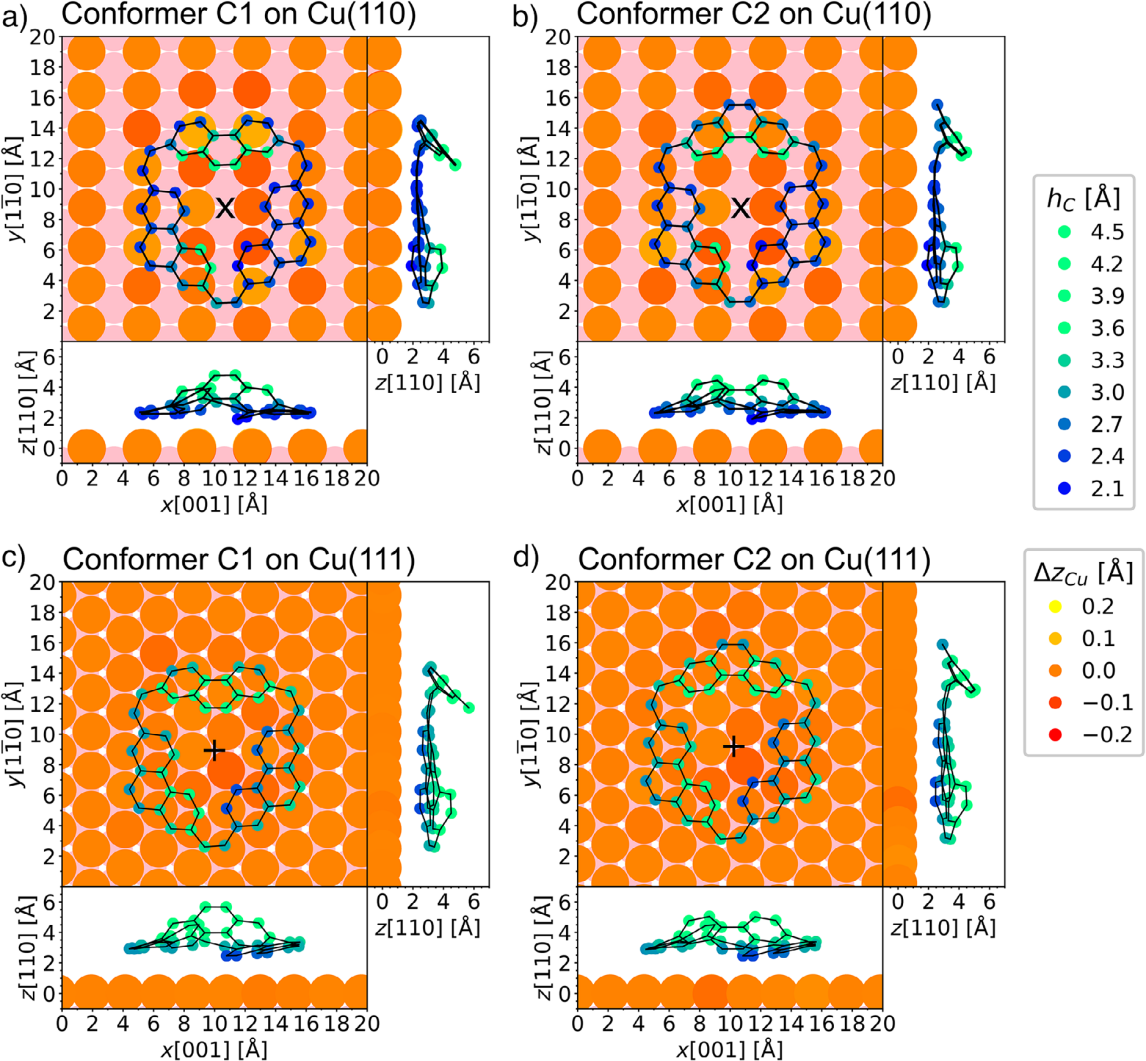
Calculated adsorption geometries of the precursor **1** as a) conformer **C1** and b) conformer **C2** at the long bridge site of Cu(110), as well as c) conformer **C1** and d) conformer **C2** at the hcp‐hollow site of Cu(111). The calculations were performed using four‐layer slabs. The color scales show the adsorption height of the carbon atoms *h_C_* (with respect to the center of the topmost copper layer) in blue to green and the relaxation of the top layer of copper atoms *Δz_Cu_* in red to yellow. A second layer of copper atoms is displayed in light pink. For clarity, only one molecule is shown for each geometry, whereas those from neighboring unit cells are omitted. The PBE‐GGA functional combined with Grimme D3 dispersion corrections^[^ ^48^ ^]^ was used.

To shed light on the origin of the high surface‐structure selectivity, the precursor was investigated by the same theoretical approach as applied to the products above. The two conformers, **C1** and **C2**, were studied on Cu(110) and compared to the same conformers adsorbed on Cu(111), which had not been investigated previously. On both surfaces, four possible adsorption sites were considered. All resulting energies are given in Table S2 in the Supporting Information. On Cu(111), the calculated energies do not indicate a significant preference for a specific adsorption site, whereas a preference for the long bride site on Cu(110) is observed for both conformers. The resulting geometries of **C1** and **C2** in the long bridge site on Cu(110) and the hcp‐hollow site on Cu(111) are presented in Figure 5. For Cu(111), the energy difference between the conformers **C1** and **C2** is negligible across all four adsorption sites which were considered. In contrast, at the preferred adsorption site on Cu(110), conformer **C1** is expected to be more favorable with an energy difference of 0.26 eV compared to conformer **C2**. Comparing the height of the carbon atoms with respect to the topmost copper atoms (as indicated by the blue color scale) in the DFT geometries (see Figure 5a,b) to the geometries on Cu(111) (see Figure 5c,d), one notices lower adsorption heights on the Cu(110) surface. This again indicates a stronger interaction between the molecules and the more open Cu(110) surface, consistent with the findings for the product species.

As shown in Scheme 1, the adsorption conformation **C1** of the precursor **1** could facilitate the activation of the C─H bonds marked by cyan circles leading to the formation of isokekulene. Conversely, the adsorption conformation **C2** of the precursor **1** could facilitate the activation of the C─H bonds marked by green circles, leading to kekulene.

**Figure 6:**
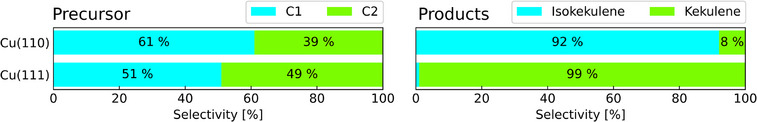
Diagram showing the relative abundances of (left) the two conformers of the precursor and (right) the products at monolayer coverage on both surfaces. The values are based on the statistical analysis presented in the Supporting Information, Figures S1 to S3.

Interestingly, the Cu(111) surface does not show any significant preference for one of the adsorption conformations in the experiment (see Figure 1c, Figure S3 for an example of the identification of the two conformers, as well as Figure 6 for an overview over the statistics). This is in agreement with their comparable energies resulting from the DFT calculation (see above). In contrast, adsorption on Cu(110) slightly favors conformer **C1** with a percentage of 61% compared to 39% adsorbed as conformer **C2** in the experiment (see Figure S2 for example images), again in agreement with the slight preference for **C1** predicted by the DFT.

Overall, comparing the distributions of the precursor conformers and the products in the monolayer as visualized in Figure 6, it becomes clear that the adsorption conformation of the precursor alone cannot explain the observed large selectivities for the formation of kekulene on Cu(111) and isokekulene on Cu(110). Notably, no significant desorption was observed on either surface during the annealing step (compare for example Figure 2a to Figure 3a). Additionally, full monolayer precursor coverages yielded full monolayers of product molecules, kekulene on Cu(111) (Figure 1d) and predominantly isokekulene on Cu(110) (Figure 1b). Thus, preferential desorption of one of the conformers cannot account for the observed selectivities either.

We, therefore, conclude that the precursor molecules originally adsorbed in the **C1** conformation on Cu(111) must be able to change their conformation in such a way that they still react to kekulene. Likewise, most precursor molecules originally adsorbed in the **C2** conformation on Cu(110) must react in such a way that isokekulene can be formed. Thus, overall, the energetics of the final products on the respective surfaces may play a significant role for the selectivity.

At first glance, a comparison of the adsorption energies per unit cell at the long bridge site on Cu(110) (Table S1, column 6), might suggest that the formation of kekulene is energetically favorable over the formation of isokekulene. However, because the two molecules occupy different surface areas, the adsorption energies per surface area are a more appropriate metric for assessing the stability of the (iso)kekulene monolayer. With approximately −4 eV nm^−2^ and −3.6 eV nm^−2^ for isokekulene in the up and down configuration, respectively, both are more stable than kekulene with approximately −3 eV nm^−2^. This higher stability of isokekulene may be an important driving force for the precursor to change its conformation and react to isokekulene, even when it is initially adsorbed as conformer **C2**, thus determining the overall selectivity for the formation of the isokekulene monolayer.

In general, while the products show a preferred orientation and adsorption site (Figure 1b,d) on both surfaces, the precursor is always found in a disordered fashion (Figure 1a,c), indicating significant molecular mobility during the reaction. As a result, possible intermediates could not be identified by means of high‐resolution STM at low temperatures. A more detailed theoretical investigation of the reaction mechanism and possible intermediate states is hindered by the large unit cell, as well as the mobility and flexibility of the precursor. The large number of possible sites and configurations would have to be reduced so drastically that meaningful and reliable results could not be obtained.

## Conclusion

In conclusion, we have observed surface structure‐dependent selectivity in intramolecular on‐surface C─H bond activation, resulting in the exclusive formation (>99%) of kekulene on Cu(111) and the high‐coverage formation of predominantly the novel cycloarene isokekulene (92%) on Cu(110), both from the same single precursor molecule. The reaction to the respective products is facilitated by the significant mobility and flexibility of the precursor. Unlike kekulene, isokekulene is nonplanar and assumes two different adsorption configurations on Cu(110), as was confirmed by scanning probe microscopy and density‐functional theory calculations. The short molecule‐surface bond length found on Cu(110) indicates a much stronger adsorbate‐substrate interaction compared to Cu(111). Overall, we attribute the high selectivity of the cyclodehydrogenation reaction to the specific energetics and stability of the reaction products on the different copper surfaces. These findings provide an effective, alternative approach for the synthesis of novel cycloarenes that are currently inaccessible via conventional solution‐based chemistry.

## Materials and Methods

The Cu(111) and Cu(110) single crystals with a miscut of less than 0.1° were purchased from MaTecK GmbH, Germany. The surfaces were prepared by cycles of Ar^+^ ion bombardment and annealing to 850 K. The macrocyclic nonplanar precursor molecule 1,4,7(2,7)‐triphenanthrena‐cyclononaphane‐2,5,8‐triene (precursor **1**) was prepared and purified in a four‐step solution‐based synthesis starting from 9,10‐dihydrophenanthrene as reported previously.^[^ ^28^ ^]^ It was evaporated in ultra‐high vacuum (base pressure 1 × 10^−10^ mbar) from a home‐built Knudsen cell or a commercial evaporator from Kentax at 500 K.

High‐resolution STM measurements (Figures 2 and 3) were performed on a commercial Scienta Omicron LT‐STM (Gen. III), which is liquid‐helium cooled to 4 K. The tungsten STM tip was mounted on a commercial qPlus sensor and decorated by a CO molecule picked up from the Cu surface. Gwyddion^[^ ^49^ ^]^ and WSxM^[^ ^50^ ^]^ were used for image processing. Additional STM measurements (Figure 1) were performed on a commercial SPECS STM Aarhus 150 STM at 100 K. Moderate filtering (Gaussian smooth, background subtraction) was applied for noise reduction.

All DFT calculations were performed within a repeated‐slab approach employing the Vienna ab initio simulation package (VASP) and using the projector augmented wave method to treat the core electrons.^[^ ^43^, ^44^, ^45^, ^46^ ^]^ The Cu(110) substrate was modelled by four‐ and five‐layer copper slabs using the theoretical lattice parameter of 3.6 Å as obtained by the PBE‐GGA functional combined with Tkatchenko–Scheffler^[^ ^47^ ^]^ as well as Grimme D3^[^ ^48^ ^]^ dispersion corrections. For the adsorption of isokekulene on Cu(110) as well as the precursor molecule, we used the experimentally observed surface unit cell described by the epitaxial matrix $\left(\begin{array}{cc} 4 & 3\\ 0 & 6 \end{array}\right)$, while for the adsorption of kekulene we resorted to the somewhat larger $\left(\begin{array}{cc} 5 & 0\\ 0 & 7 \end{array}\right)$ overlayer to properly fit the molecule into the cell. We considered the following adsorptions sites: top, hollow, short bridge, and long bridge with respect to the central void of (iso)kekulene as well as the precursor on Cu(110) and top, bridge, hollow‐hcp, and hollow‐fcc for the precursor on Cu(111). Geometry relaxations were performed including the two top‐most copper layers. Simulated STM images (Figure 3c,d) were obtained using the Tersoff−Hamann method.^[^ ^51^ ^]^

## Supporting Information

Supporting information available on the statistics of all species in STM, a LEED measurement, and energies resulting from the DFT calculations.

## Conflict of Interests

The authors declare no conflict of interest.

## Supporting information



Supporting Information

## Data Availability

The data that support the findings of this study are available from the corresponding author upon reasonable request.
