# Supplementary material for: Highly Structure‐Selective On‐Surface Synthesis of Isokekulene Versus Kekulene
Source: Angew Chem Int Ed Engl. 2025 Jul 29;64(36):e202509932. doi: 10.1002/anie.202509932 (PMC12402830; doi:10.1002/anie.202509932)
Supplement: Supplementary file 1 — Supporting Information [file ANIE-64-e202509932-s001.pdf]

Supporting Information

to

Highly Structure-Selective On-Surface Synthesis of Isokekulene versus Kekulene

Zilin Ruan,[†,1] Qitang Fan,[†,1,2] Alexander Reichmann,[3,‡] Faming Kang,[1] Tim Naumann,[1] Simon Werner,[1] Olaf Kleykamp,[1] Jose Martinez-Castro,[4,5,6] Felix Lüpke,[4,5,7] Anja Haags,[4,5,8] François C. Bocquet,[4,5] Christian Kumpf,[4,5,8] Serguei Soubatch,[4,5] Jörg Sundermeyer,[1] Peter Puschnig,[3] F. Stefan Tautz,[4,5,8] J. Michael Gottfried,[1] and Sabine Wenzel[*,1,4,5]

[†]Both authors contributed equally.

1 Department of Chemistry, Marburg University, 35037 Marburg, Germany
2 Hefei National Research Center for Physical Sciences at the Micro scale, Synergetic Innovation Center of Quantum Information & Quantum Physics, and New Cornerstone Science Laboratory, University of Science and Technology of China, Hefei, Anhui 230026, China
3 Institute of Physics, University of Graz, NAWI Graz, 8010 Graz, Austria
4 Peter Grünberg Institute (PGI-3), Forschungszentrum Jülich, 52425 Jülich, Germany
5 Jülich Aachen Research Alliance (JARA), Fundamentals of Future Information Technology, 52425 Jülich, Germany
6 Experimental Physics II B, RWTH Aachen University, 52074 Aachen, Germany
7 II. Physikalisches Institut, Universität zu Köln, 50937 Köln, Germany
8 Experimental Physics IV A, RWTH Aachen University, 52074 Aachen, Germany
[‡]Current Address: Chair of Physical Metallurgy, University of Leoben, 8700 Leoben, Austria

*E-mail: sabine.wenzel@uni-marburg.de

S.1. STM Images for Statistical Evaluation

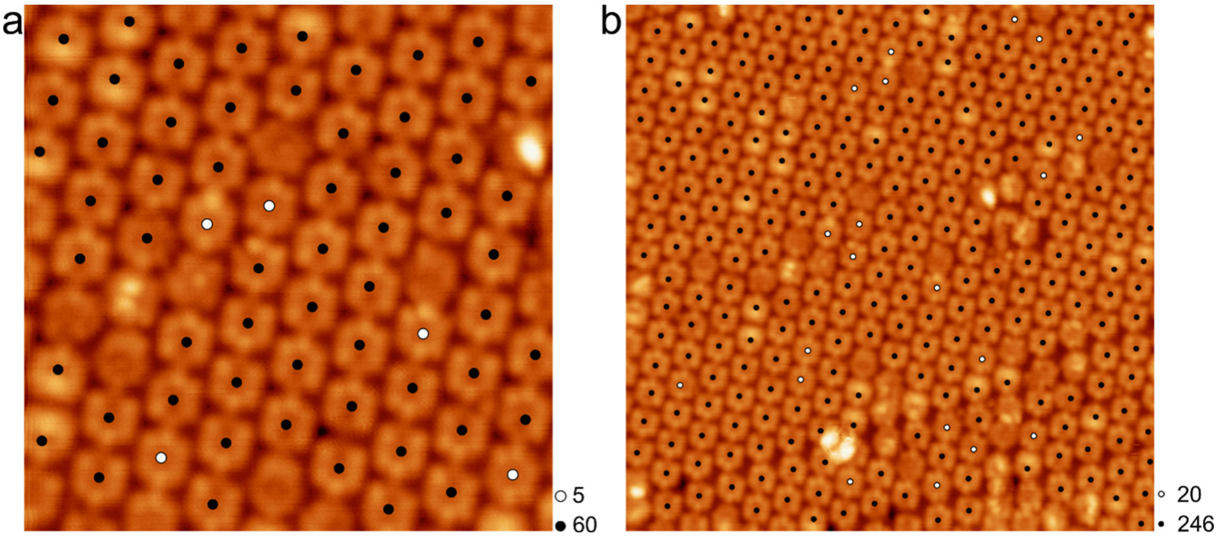

Figure S1: Two distinct STM images recorded under identical conditions after deposition of a monolayer of precursor **1** onto Cu(110) at 300 K and subsequent annealing to 500 K. The formed isokekulene and kekulene molecules have been marked with black and white dots, respectively. A ratio of 92 % isokekulene : 8 % kekulene is obtained. Sizes of the images are (a) 15 nm x 15 nm and (b) 30 nm x 30 nm.

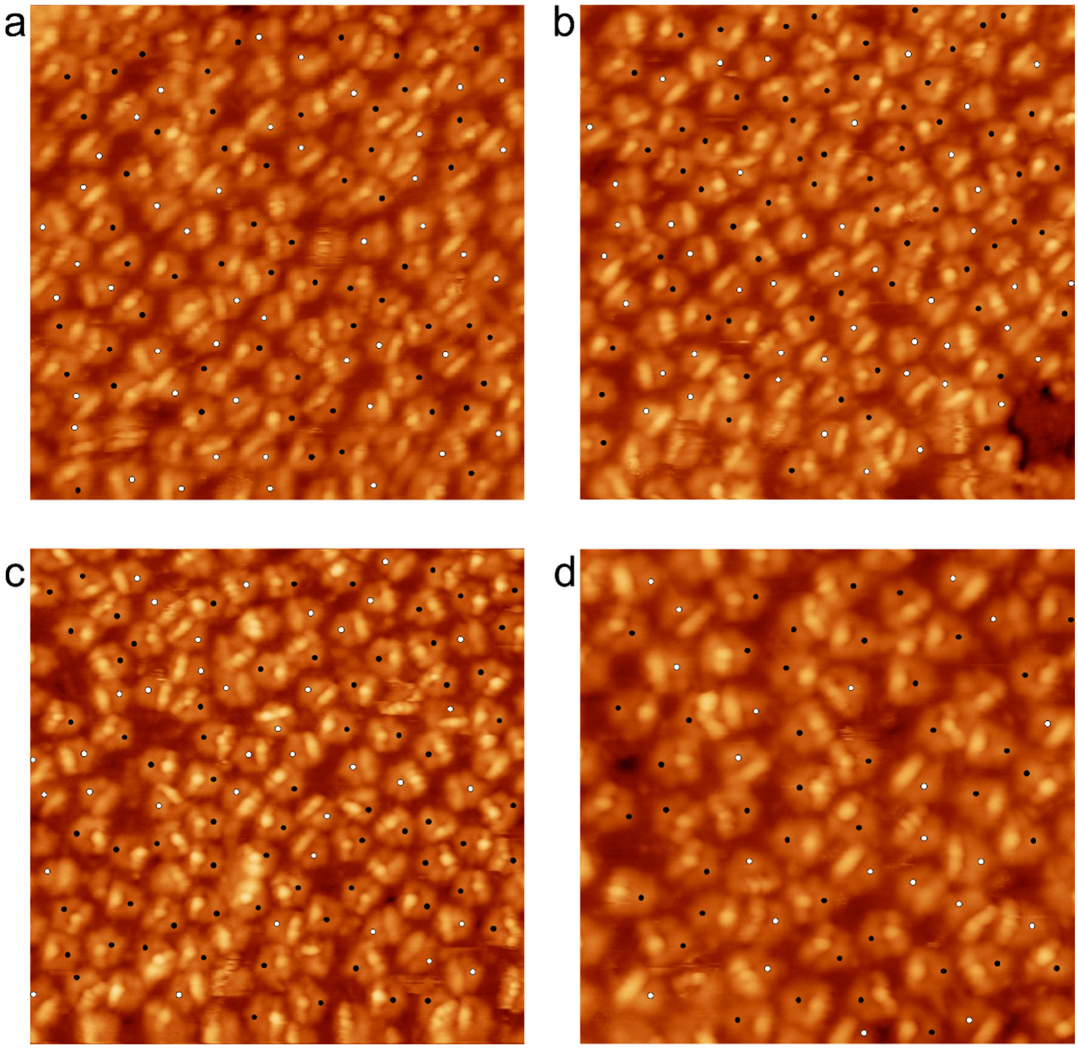

Figure S2: Four distinct STM images recorded under identical conditions after deposition of a monolayer of precursor 1 onto Cu(110) at 300 K. The two different adsorption conformations (**C1** and **C2**) of precursor 1 on Cu(110) are marked with black and white dots, respectively. A ratio of 61 % **C1** : 39 % **C2** is obtained. Sizes of the images are (a-c) 20 nm × 20 nm and (d) 15 nm × 15 nm.

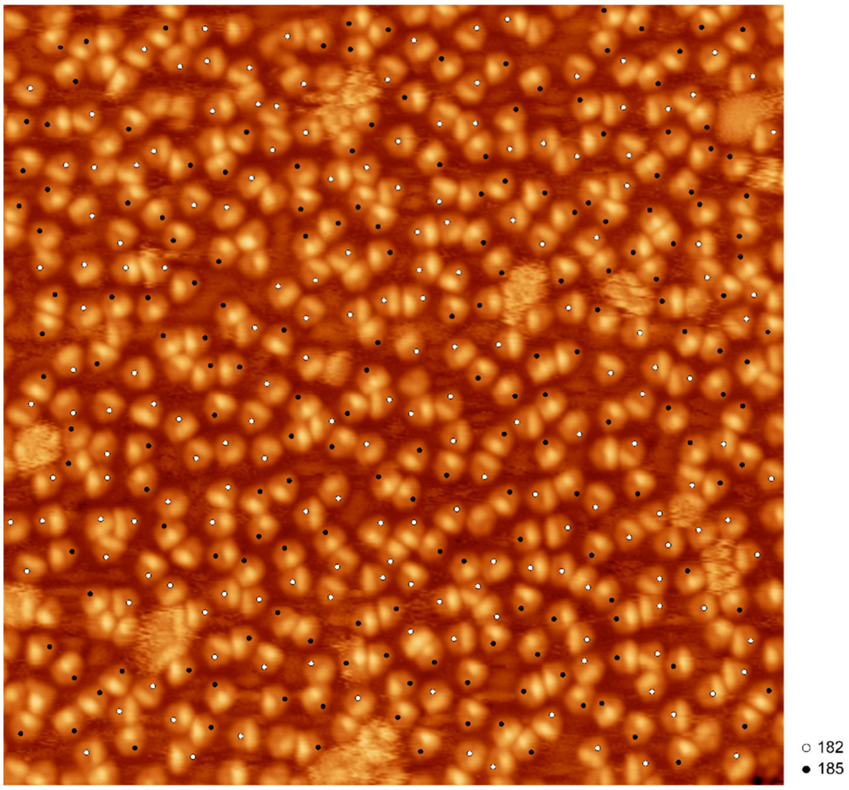

Figure S3: Overview STM image of a full monolayer of the precursor 1 on Cu(111) as presented in Figure 1c in the main text. The two different adsorption conformations (**C1** and **C2**) of precursor **1** on Cu(110) are marked with black and white dots, respectively. A ratio of 50 % **C1** : 50 % **C2** is obtained. The tunneling parameters are *U* = 3.0 V, *I* = 0.08 nA and the size of the image is 50 nm x 50 nm.

S.2. Low-Energy Electron Diffraction

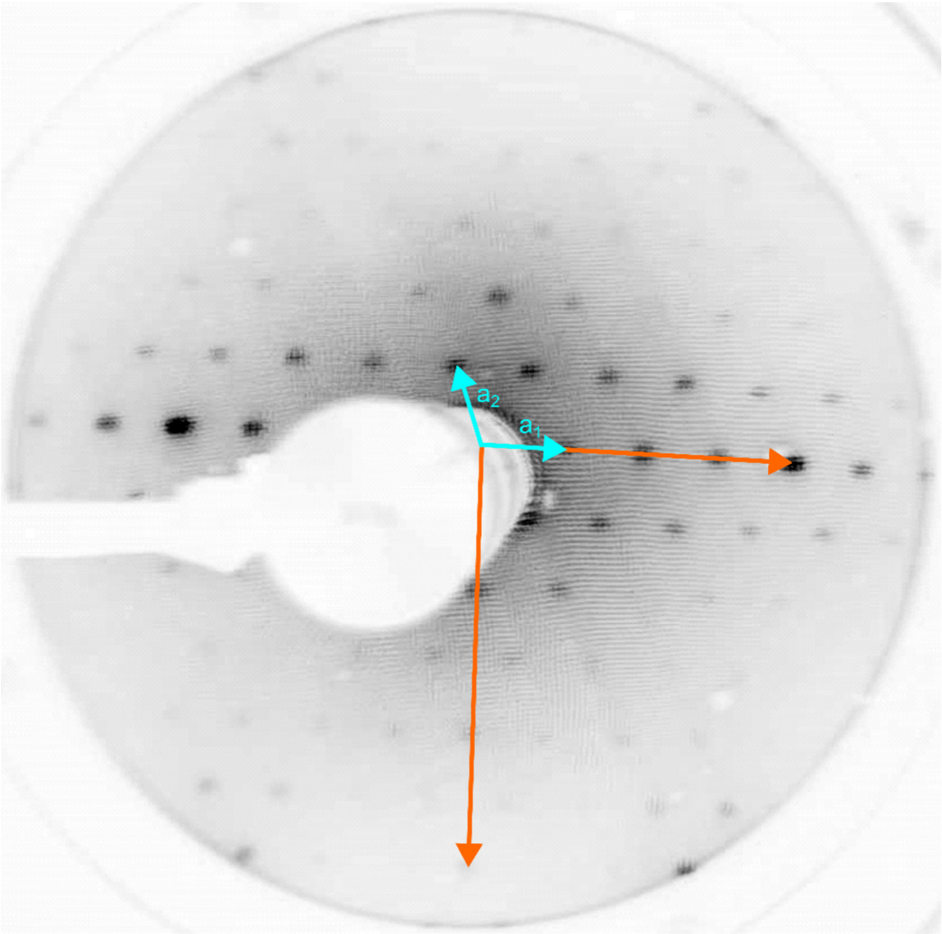

Figure S4: LEED pattern of a monolayer of isokekulene (containing a small concentration of kekulene, see Figure 1b) on Cu(110) measured with an electron energy of 45 eV. The unit cell parameters of the isokekulene monolayer structure were determined to a_1 = 1.6 nm and a_2 = 1.5 nm, and γ = 118° (see cyan arrows). The substrate unit cell marked in orange was used for calibration.

S.3. Density Functional Theory

Table S1: DFT results for kekulene and isokekulene at different adsorption sites on Cu(110). $\bar{h}_C$ is the average adsorption height of the carbon atoms with respect to the center of the topmost copper layer. Total energies E_{total} are expressed in eV per unit cell. For the most stable adsorption sites (bold), the resulting geometries are displayed in Figure 4 in the main text. For the other sites, ΔE_{total} gives the energy difference with respect to the most stable site. Adsorption energies were calculated as $E_{ad} = E_{total} - (E_{molecule} + E_{surface})$ from the energies of the separate molecule $E_{molecule}$ and the bare surface $E_{surface}$ (with both the molecule as well as the surface in the geometry found for the combined system). To allow for comparison of the different molecules, the adsorption energies are given per unit cell (last but one column) as well as per surface area (last column).

Molecule	Adsorption Site	$\bar{h}_C$ [Å]	E_{total} [eV]	ΔE_{total} [eV]	E_{ad} [eV]	E_{ad} [eV/nm^2]
kekulene	top	2.54	−1185.21	1.28	−7.94	−2.55
	short bridge	2.46	−1185.82	0.67	−8.55	−2.74
	**long bridge**	**2.41**	**−1186.49**	**0**	**−9.22**	**−2.96**
	hollow	2.48	−1185.98	0.51	−8.71	−2.79
isokekulene up	top	2.69	−979.38	1.09	−7.62	−3.56
	short bridge	2.59	−979.76	0.71	−8.00	−3.74
	**long bridge**	**2.48**	**−980.47**	**0**	**−8.71**	**−4.07**
	hollow	2.57	−980.05	0.42	−8.29	−3.88
isokekulene down	top	2.87	−978.87	1.44	−6.78	−3.17
	short bridge	2.87	−978.97	1.34	−6.88	−3.22
	long bridge	2.67	−979.79	0.52	−7.70	−3.60
	**hollow**	**2.6**	**−980.31**	**0**	**−8.22**	**−3.84**

Table S2: DFT results for the conformers **C1** and **C2** of the precursor at four different adsorption sites each on Cu(110) and Cu(111). Total energies E_{total} are expressed in eV per unit cell. For the most stable adsorption sites (bold), the resulting geometries are displayed in Figure 5 in the main text. For the other sites, ΔE_{total} gives the energy difference with respect to the most stable site. $E_{C1} - E_{C2}$ is the energy difference between the two adsorption conformers at each site.

Conformer	Adsorption Site	E_{total} [eV]	ΔE_{total} [eV]	$E_{C1} - E_{C2}$ [eV]
C1 on Cu(110)	top	−1540.51	1	−0.04
	short bridge	−1540.54	0.98	0.21
	**long bridge**	**−1541.52**	**0**	**−0.26**
	hollow	−1541.43	0.09	−0.29
C2 on Cu(110)	top	−1540.47	0.78	
	short bridge	−1540.75	0.51	
	**long bridge**	**−1541.25**	**0**	
	hollow	−1541.14	0.12	
C1 on Cu(111)	top	−1795.98	0.35	0.03
	bridge	−1796.25	0.08	0.02
	**hollow-hcp**	**−1796.33**	**0**	**0**
	hollow-fcc	−1796.29	0.04	0.01
C2 on Cu(111)	top	−1796.01	0.32	
	bridge	−1796.27	0.06	
	**hollow-hcp**	**−1796.32**	**0**	
	hollow-fcc	−1796.30	0.03	
